# Fostering mask-wearing with virality metrics and social media literacy: evidence from the U.S. and Korea

**DOI:** 10.3389/fpsyg.2023.1151061

**Published:** 2023-05-24

**Authors:** Dam Hee Kim, Ozan Kuru, Jiaqi Zeng, Seongcheol Kim

**Affiliations:** ^1^Department of Communication, The University of Arizona, Tucson, AZ, United States; ^2^Department of Communications and New Media, National University of Singapore, Singapore, Singapore; ^3^School of Media and Communication, Korea University, Seoul, Republic of Korea

**Keywords:** mask-wearing, social media literacy, virality metrics, opinion climate, perceived norms, social media engagement, public health campaign

## Abstract

Although social media can pose threats to the public health by spreading misinformation and causing confusion, they can also provide wider access to health information and opportunities for health surveillance. The current study investigates the ways in which preventive health behaviors and norms can be promoted on social media by analyzing data from surveys and experiments conducted in the U.S. and South Korea. Survey results suggest that the pathway from social media use for COVID-19 information to mask-wearing behavior through mask-wearing norms emerges only among individuals with strong perceived social media literacy in the U.S. Experimental findings show that wear-a-mask campaign posts on social media foster mask-wearing norms and behavioral intention when they come with large (vs. small) virality metrics (e.g., Likes, shares) in both the U.S. and South Korea. Additionally, American users are more willing to engage with posts that come with supportive (vs. mixed) comments by Liking, sharing and commenting. The results highlight the need to cultivate social media literacy and opportunities for exploiting social media virality metrics for promoting public health norms and behaviors.

## 1. Introduction

People increasingly use social media for health information (Chou et al., 2021), including COVID-19 news (Mitchell and Liedke, 2021). While social media use for health can be beneficial in terms of increased access to health information and opportunities for health surveillance, it can also pose challenges involving confusion, loss of trust in experts, information overload and misinformation (Moorhead et al., 2013; Huber et al., 2019). The amount of misinformation about COVID-19 was particularly high on social media compared to other digital platforms (e.g., Kouzy et al., 2020). Various content on social media, ranging from original posts and viral hashtags to user comments and endorsements, produces a heterogeneous information environment. Considering that inaccurate health information can be presented on equal footing along with guidance from health authorities on social media, the current study examines whether preventive health behaviors and norms can be fostered on social media through organic interactions emerging between public health posts by institutions such as C.D.C. and user engagement these posts generate. This question is an important one to answer during global health crises such as the COVID-19 pandemic; as efforts to limit the spread of the coronavirus and protect vulnerable segments largely depend on individuals’ willingness to participate in preventive and hospitals health behaviors (e.g., mask-wearing, Lewandowsky and Van Der Linden, 2021), especially in contexts such as public transportation and hospitals. Continued mask wearing, particularly in closed social contexts, continues to be an important recommendation 3 years into the pandemic to protect vulnerable people (World Health Organization, 2022).

To investigate how mask-wearing norms and behaviors operate and can be promoted on social media, the current study analyzes data from surveys (Study 1) and experiments (Study 2) conducted in the U.S. and South Korea. Study 1 establishes a pathway from social media use for COVID-19 information to mask-wearing behavior through mask-wearing norms by drawing from the Theory of Reasoned Action and Planned Behavior (Ajzen, 1991). Importantly, we test whether this pathway is moderated by *perceived social media literacy*, which reflects users’ ability to critically evaluate and process misinformation (Borah, 2022; see also Li et al., 2020 for the link between perceived and actual literacy). Next, focusing on the potential of health campaigns on social media involving heightened interactivity (Chou et al., 2013), Study 2 examines if mask-wearing norms and behavioral intention can be fostered upon exposure to wear-a-mask social media posts by health authorities, the Centers for Diseases Control and Prevention (CDC) in the U.S. and the Disease Control and Prevention Agency (DCPA) in Korea. As these institutional posts generate user reactions, the experiments also test if mask-wearing norms and behavioral intention vary by the campaign posts’ differing user interaction factors: *virality metrics* (high vs. low engagement in the form of number of Likes, comments and shares) and *opinion climate* (supportive, including only pro-mask sentiments vs. mixed comments, including both pro- and anti-mask sentiments).

**Figure 1:**
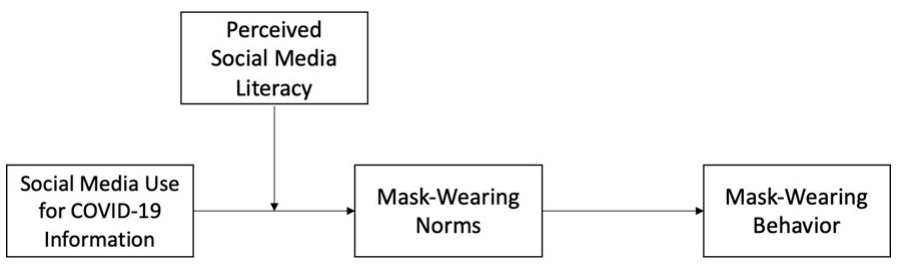
Study 1: the theoretical model.

Overall, Study 1 establishes a pathway from social media use for COVID-19 information to mask-wearing norms and behavior, which emerges only among people with strong perceived social media literacy (Figure 1) in the U.S. Study 2 reports experimental evidence that wear-a-mask campaign messages on social media strengthen mask-wearing norms and behavioral intentions if they come with large virality metrics. This study seeks to contribute to the prior scholarship in the following ways: First, it clarifies paths through which social media can limit or facilitate health preventive behaviors and normative perceptions. The mechanisms involve perceived social media literacy and messages by health authorities that come with large virality metrics rather than supportive comments. Second, in doing so, this study utilized both survey and experimental methods in an effort to ensure both internal and external validity of the findings. By incorporating how institutional and general user interactions might shape individuals’ perceived norms, the experiment provides a more realistic testing of social media effects on public health, Finally, the current study collected data from two different contexts in terms of the pandemic experience and cultures, the U.S. and South Korea (Bromwich, 2020).

## 2. Study 1

### 2.1. Social media use, literacy, and normative perceptions and behavior

There are various considerations on how social media use for COVID-19 news and information can shape normative perceptions and health behaviors. On the one hand, there is a lot of health misinformation on social media both in general and in the context of COVID-19 (Al Khaja et al., 2018; Kouzy et al., 2020) and this misinformation is associated with less frequent health protective behaviors such as vaccination (Pluviano et al., 2017; Romer et al., 2022). In the context of mask wearing, there are mixed results. Misinformation about mask wearing, such as that it actually increases COVID-19 exposure levels, has been widespread especially earlier in the pandemic (Newswise, 2020). Yet, research also showed that social and mobile media use was associated with increased incidences of mask wearing (Liu, 2020). According to Hornik et al. (2021), misinformation about COVID-19 also did not predict mask wearing reduction when specific beliefs about mask wearing were accounted for. This leads us to consider the heterogeneous information environment on social media above and beyond the prevalence of misinformation. Moreover, mask wearing norms could be shaped by not just the amount of reliable vs. misleading information, but a number of other factors on social media such as different actors and user engagement metrics.

Different platforms, nature of content, and sources of information on social media create a heterogenous information environment. On the one hand, platforms differ in the prevalence of pro-mask content. A study of Twitter discourse in the U.S. during the first year of the pandemic found that most discussions were composed of pro-mask tweets, although there were anti-vaccine groups as well (Lang et al., 2021). On the other hand, numerous studies showed the prevalence of anti-mask comments (Keller et al., 2021) and groups on Facebook (Ayers et al., 2021). Aside from the platform differences, the nature of pro- and anti-mask posts on social media differed in a number of ways as well. For example, on Twitter, posts with anti-mask sentiments were found to include more uncivil and toxic language (Pascual-Ferrá et al., 2021). Next, the sources of social media content also complicate the picture: Institutional pages and accounts of health authorities, such as the World Health Organization, CDC and Ministry of Health across nations campaigned for mask wearing for most of the pandemic. Social media companies themselves also tested messages for mask wearing norms; exposure to the campaign messages increased the frequency of self-reported mask wearing (Chen and Sullivan, 2021).

However, norm perceptions could be further shaped by popularity metrics on social media. These would involve both comments and expressions such as Likes and endorsements, which are key actions through which people interact on social media. A study analyzing comments to the CDC posts encouraging mask wearing revealed diverging perceptions; some people resisted these messages and expressed anger in comments, particularly early in the pandemic when there was inconsistent messaging due to limited access to masks (Batova, 2022). Also, content from individual users and celebrities can be much more mixed, with conservative influencers and celebrities posting against mask wearing and regulations, particularly in more polarized countries like the U.S. For example, pro- and anti- mask hashtags on Twitter polarized the issue along partisan lines especially early in the pandemic (Lang et al., 2021). This makes social media a mixed information environment where both anti- and pro-mask content and opinions were present throughout the pandemic. Accordingly, we ask:

*RQ1*: What is the relationship between social media use for COVID-19 information and mask-wearing norms?

While we investigate the relationship between social media COVID-19 information exposure and normative perceptions on mask wearing as a research question, we expect to see differences in this relationship based on individuals’ levels of social media literacy. In other words, social media use and social media literacy would operate together to form normative perceptions about mask wearing. Literacy, overall, is an important factor in shaping how information is processed. Greater media and news literacy has been associated with more critical and mindful information processing (Tully et al., 2020; Vraga et al., 2021) although it may also be associated with stronger skepticism (Maksl et al., 2015). Individuals with greater news literacy were found to be less reliant on endorsement metrics such as the number of comments and likes when evaluating information; they instead focused on content and titles of news more (Tully, 2021). In the context of identifying fake information, a study examined four types of literacy –media, information, digital, and news literacy, to find that only information literacy had a significant positive association (Jones-Jang et al., 2021). While these studies suggest a somewhat mixed picture, the stronger trend in the results is that overall media literacy is associated with enhanced scrutiny, attentiveness and information processing. Yet, the role of social media literacy, has not been examined to date, particularly in the context of whether it can moderate the association between social media use and mask wearing norms.

In the related context of health literacy, trust in social media content of health professionals such as doctors was an important factor for the adoption of preventative behaviors such as mask wearing (Niu et al., 2021). Recent research shows that individuals are responsive to social endorsements in the form of Likes on Facebook, and perceive posts by health authorities with greater endorsement as more credible (Borah and Xiao, 2018). Individuals who perceive they have stronger social media literacy may be more responsive to these indicators, and hence, they would display a stronger association between their social media use and perceived norms about mask wearing. Thus, while the informational and normative content on social media is heterogeneous, those individuals with greater perceived social media literacy would a) be more critical in processing the diverse information they are exposed to and b) be more responsive to social signals communicated on the platform, such as the popularity and prevalence of mask wearing content by others.

*H1*: The relationship between social media use for COVID-19 information and mask-wearing norms will be positive for individuals with the highest levels of perceived social media literacy. The relationship will decline in magnitude as perceived social media literacy decreases.

### 2.2. Normative perceptions and behavior

As a key predictive factor of health behavior according to the Theory of Reasoned Action and Planned Behavior (Ajzen, 1991), perceived norms play a sizable and significant role in health behavior in general, as reviews show (Miller and Prentice, 2016). Surveys showed that social influence increases mask wearing willingness in various countries, including China (Wang et al., 2021) and South Africa (Burger et al., 2022). Numerous experimental interventions aimed to manipulate perceived norms either directly or indirectly in order to boost mask wearing. For example, providing “information about how masks protect others increases the likelihood that someone would wear a mask or encourage others to do so,” but these effects are not consistent across countries (Bokemper et al., 2021, p. 1). Also, promotion of altruism was found to increase mask wearing while social shaming did not predict any increase in the U.S. (Bir and Widmar, 2021). Aside from interventions for voluntary behavior change, legislation also helps solidify mask wearing norms. Breaking mask rules in public settings is not only a normative issue but a legal one as well, which can closely shape norms in turn. An analysis of 38 countries found that mask mandates significantly and substantially increased mask wearing during the pandemic (Badillo-Goicoechea et al., 2021). In the U.K., legislation about social distancing during COVID-19 shaped norm perceptions (Galbiati et al., 2021). Given these multiple considerations, we expect that greater perceived norms regarding mask wearing will predict similar behavioral adoption.

*H2*: Mask-wearing norms will positively predict mask-wearing behavior.

Taking H1 and H2 together, we finally advanced H3 about the moderated mediation relationship (Figure 1). Building on the same logic that individuals with greater social media literacy would be more critical in information processing and responsive to social signals, we expect that social media literacy should also moderate the indirect relationship between social media use and mask wearing behaviors through the mediating role of norms.

*H3*: The indirect relationship between social media use for COVID-19 information and mask-wearing behavior through mask-wearing norms will be positive for individuals with the highest levels of perceived social media literacy. The relationship will decline in magnitude as perceived social media literacy decreases.

### 2.3. Materials and methods

#### 2.3.1. Sample

The current study analyzed data from two online national surveys, the first one conducted in the U.S. between June 15 and 30, 2021 (*N* = 1,194) and the second one in South Korea between September 3 to 6, 2021 (*N* = 550).^1^ Research companies, Dynata and Macromill Embrain, were contracted for data collection through their online panels in the U.S. and South Korea, respectively. To ensure that the samples resembled the American and Korean populations, demographic quotas were applied for age, gender, household income and region. The U.S. sample consisted of 45.4% males, 43.7% females and 0.2% of those who identified as ‘other,’ with a mean age of 46.8 years. The Korean sample included 49.1% of males and 50.9% of females, with a mean age of 44.7 years. The surveys were conducted in English and Korean, respectively.

#### 2.3.2. Measures

##### 2.3.2.1. Social media use for COVID-19 information

Respondents were asked, “In the past 14 days, on social media, how often they: (a) read or watched content about COVID-19 that people shared, (b) read or watched content about COVID-19 from news sources or public figures that they followed; and (c) read people’s personal opinions about COVID-19.” Response options ranged from 1 (never) to 6 (every day). An index was created by averaging the three items (U.S.: *M* = 2.89, *SD* = 1.68, *α* = 0.93; Korea: *M* = 4.92, *SD* = 0.31, *α* = 0.84).^2^

##### 2.3.2.2. Perceived social media literacy

Respondents indicated how true the following two statements about their social media use were: “I can easily spot false information on social media” and “I have the necessary skills to check the accuracy of information that I receive on social media” (adapted from Borah, 2022). Response options ranged from 1 (not at all true) to 5 (extremely true). An index was created by averaging the two items (U.S.: *M* = 3.41, *SD* = 1.07, *r* = 0.67; Korea: *M* = 2.67, *SD* = 0.89, *r* = 0.69).

##### 2.3.2.3. Mask-wearing norms

Respondents indicated how much they disagreed or agreed with the following two statements: “Most people important to me wear masks/face coverings” and “Most people important to me would encourage wearing masks/face coverings.” Response options ranged from 1 (strongly disagree) to 5 (strongly agree) (U.S.: *M* = 3.73, *SD* = 1.16, *r* = 0.79; Korea: *M* = 4.77, *SD* = 0.45, *r* = 0.48).

##### 2.3.2.4. Mask-wearing behavior

Respondents reported how often they wore a mask or face covering in the past 2 weeks, using a 5-point-scale, ranging from 1 (never) to 5 (always) (U.S.: *M* = 3.40, *SD* = 1.42; Korea: *M* = 4.92, *SD* = 0.31).

##### 2.3.2.5. Control variables

First, demographic variables including sex (male = 0, female/other = 1), age, income and education were controlled for. Additionally, we controlled for three variables that could influence mask-wearing norms and behavior, including political ideology, news media use, and COVID-19-related self-efficacy (Sheeran et al., 2016). *Political ideology* was measured on a 7-point-scale, ranging from 1 (extremely conservative) to 7 (extremely liberal). *News media use* for COVID-19 information was measured by asking respondents in the past 14 days, how often they used the following three sources to get news and information about COVID-19: print newspapers, online news sources and TV news and radio shows on a 6-point-scale (U.S.: *M* = 3.37, *SD* = 1.48; Korea: *M* = 4.14, *SD* = 1.02). *Self-efficacy* of preventing oneself from getting COVID-19 was measured by asking respondents how much they disagreed or agreed with: “I am confident in my COVID-19 preventive behaviors” and “I am confident that I will not be infected with COVID-19” on a 5-point scale (Niu et al., 2021) (U.S.: *M* = 3.98, *SD* = 0.73; Korea: *M* = 3.50, *SD* = 0.80).

#### 2.3.3. Analysis

To test the model (Figure 1), we used the SPSS macro PROCESS model 7 utilizing ordinary least squares regressions (Hayes, 2013).

### 2.4. Results

#### 2.4.1. The U.S.

**Figure 2:**
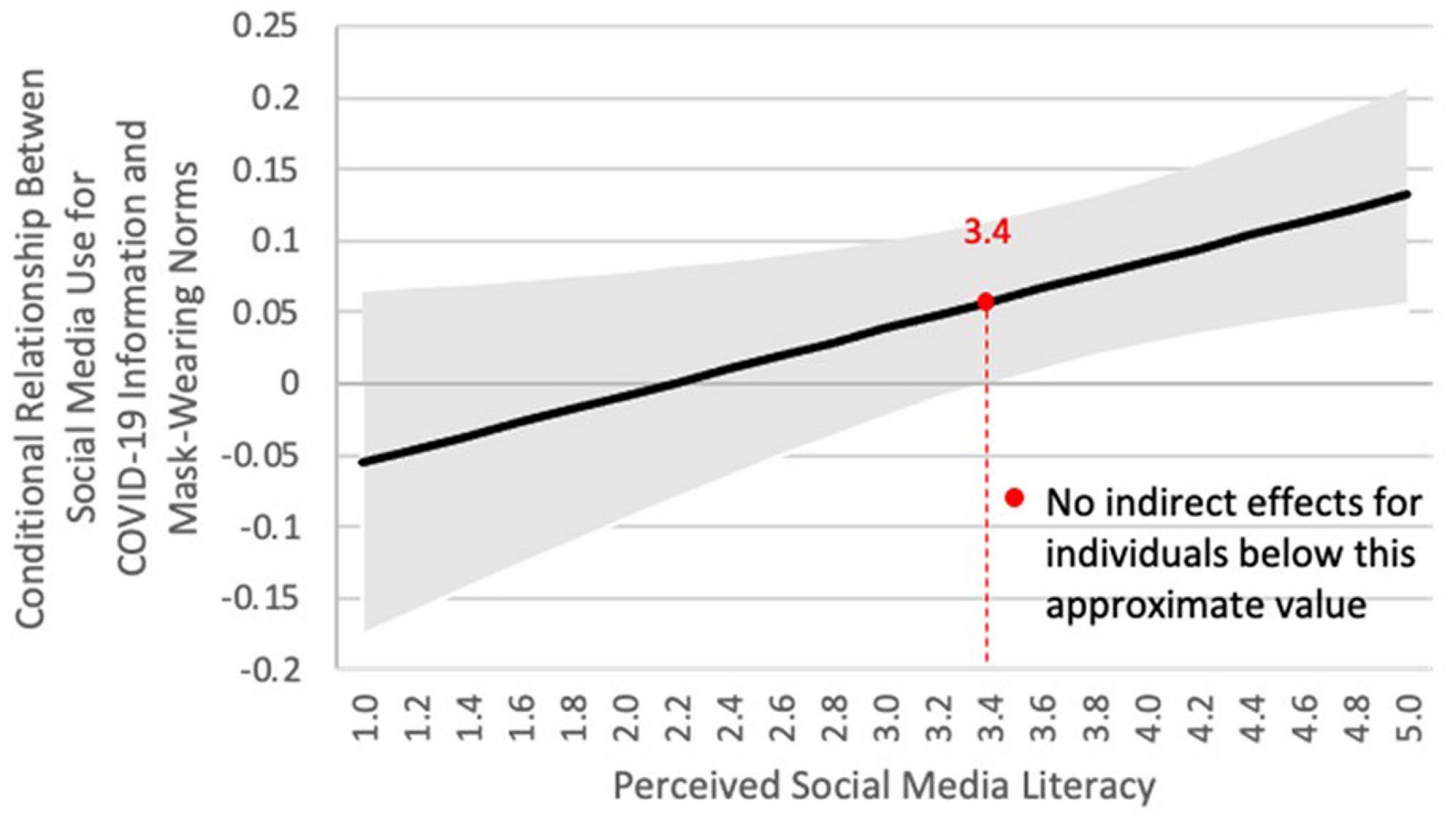
Study 1: conditional relationships between social media news use and mask-wearing behavior as a function of perceived social media literacy in the U.S.

Research question 1 investigated the relationship between social media use for COVID-19 information and mask-wearing norms. To test RQ1, mask-wearing norms were regressed on social media use while demographic variables, political ideology, news media use and self-efficacy were controlled for (Table 1, first column). Social media use for COVID-19 information was found to positively predict mask-wearing norms (*b* = 0.07, SE = 0.03, *p* < 0.05). H1 predicted that the relationship between social media use for COVID-19 information and mask-wearing norms would be positive for individuals with the highest levels of perceived social media literacy, and this relationship would decline in magnitude as perceived social media literacy decreased. To test H1, the interaction term between social media use and perceived social media literacy was added to the model (Table 1, second column). The interaction term was positive and significant (*b* = 0.05, SE = 0.02, *p* < 0.05). We probed this relationship using the Johnson-Neyman technique (Hayes, 2013). As shown in Figure 2, the conditional relationship between social media use and mask-wearing norms is positive only among individuals holding perceived social media literacy higher than 3.39. For instance, the relationship among those holding perceived social media literacy of 3.4 was *b* = 0.06 (0.03), 95% CI [0.001, 0.113]. The relationship decreased as individuals held lower perceived social media literacy. This evidence is supportive of H1.

**Table 1:** Study 1: predicting mask-wearing norms and behavior in the U.S.

	Mask-wearing norms *b* (SE)	Mask-wearing norms *b* (SE)	Mask-wearing behavior *b* (SE)
Constant	1.48 (0.26)**	1.90 (0.31)**	1.07 (0.26)**
Social media news use	0.07 (0.03)*	−0.10 (0.08)	0.12 (0.03)**
Perceived social media literacy	0.04 (0.04)	−0.06 (0.06)	–
SM news use × perceived SM literacy	–	0.05 (0.02)*	–
Mask-wearing norms	–	–	0.68 (0.03)**
Sex	−0.09 (0.07)	−0.09 (0.07)	0.16 (0.08)*
Age	0.00 (0.00)	0.00 (0.00)	−0.01 (0.00)**
Income	−0.01 (0.02)	−0.01 (0.02)	−0.03 (0.02)#
Education	0.06 (0.03)*	0.07 (0.03)**	0.00 (0.03)
Political ideology	0.13 (0.02)**	0.13 (0.02)**	0.02 (0.02)
Self-efficacy	0.10 (0.05)*	0.09 (0.05)#	−0.04 (0.05)
News media use	0.18 (0.03)**	0.18 (0.03)**	0.04 (0.03)
*R* square	0.19	0.20	0.43
df	962	961	962

**p < 0.01, *p < 0.05, #*p* < 0.10.

Next, H2 predicted that mask-wearing norms would positively predict mask-wearing behavior, and we found support for H2 (*b* = 0.16, SE = 0.03, *p* < 0.01). Finally, H3 predicted that the indirect relationship between social media use for COVID-19 information and mask-wearing behavior through mask-wearing norms would be positive for individuals with the highest levels of perceived social media literacy, and this relationship would decline in magnitude as perceived social media literacy decreased. Overall, we found support for H3, the proposed moderated mediation model, as the confidence interval did not cross zero (*b* = 0.03, SE = 0.01, 95% CI [0.004, 0.060]). To probe this relationship, we followed a pick-a-point procedure, setting up the value of the moderator, perceived social media literacy, to one standard deviation below the mean, the mean, and one standard deviation above the mean (Hayes, 2013). The relationship between social media use and mask-wearing behavior through mask-wearing norms was positive and statistically significant for individuals with a high level of perceived social media literacy of 4.48 (*b* = 0.07, SE = 0.02, 95% CI = [0.028, 0.12]) while it was not statistically significant among those with a low or medium level of perceived social media literacy (Table 2).

**Table 2:** Study 1: conditional indirect relationships between social media news use and mask-wearing behavior through mask-wearing norms at values of perceived social media literacy in the U.S.

Perceived social media literacy	Point estimate	95% C.I.
2.35 (−1SD)	0.01 (0.03)	−0.050 to 0.062
3.42 (Mean)	0.04 (0.02)	−0.002 to 0.082
4.48 (+1SD)	0.07 (0.02)	0.028 to 0.120

#### 2.4.2. South Korea

First, regarding RQ1, social media use for COVID-19 information did not appear to positively predict mask-wearing norms (*b* = 0.01, SE = 0.01, *p* > 0.05, Table 3, first column). With regard to H1, the interaction term between social media use and social media information literacy was marginally significant (*b* = 0.02, SE = 0.01, *p* < 0.10, Table 3, second column). However, further analyses using the Johnson-Neyman technique showed there were no transition points within the range of the moderator that were statistically significant (H1 rejected).

**Table 3:** Study 1: predicting mask-wearing norms and behavior in Korea.

	Mask-wearing norms *b* (SE)	Mask-wearing norms *b* (SE)	Mask-wearing behavior *b* (SE)
Constant	4.34 (0.15)**	4.55 (0.19)**	4.05 (0.17)**
Social media news use	0.01 (0.01)	−0.06 (0.04)	0.00 (0.01)
Perceived social media literacy	0.00 (0.02)	−0.07 (0.05)	–
SM news use × perceived SM literacy	–	0.02 (0.01)#	–
Mask-wearing norms	–	–	0.16 (0.03)**
Sex	−0.13 (0.04)**	−0.13 (0.04)**	0.04 (0.03)
Age	0.00 (0.00)	0.00 (0.00)	0.00 (0.00)
Income	0.03 (0.01)**	0.03 (0.01)**	0.02 (0.01)*
Education	0.02 (0.01)	0.02 (0.01)	−0.01 (0.01)
Political ideology	0.01 (0.02)	0.01 (0.02)	−0.02 (0.01)
Self-efficacy	0.11 (0.02)**	0.11 (0.02)**	0.01 (0.02)
News media use	−0.02 (0.02)	−0.02 (0.02)	0.03 (0.01)#
*R* square	0.08	0.08	0.09
df	518	517	518

**p < 0.01, *p < 0.05, #*p* < 0.10.

Next, we found support for H2 as mask-wearing norms positively predicted mask-wearing behavior (*b* = 0.16, SE = 0.03, *p* < 0.01). Finally, we did not find support for H3, the proposed moderated mediation model as the confidence interval crossed zero (*b* = 0.004, SE = 0.003, 95% CI [−0.0004, 0.009]).

## 3. Study 2

### 3.1. The influence of virality metrics on social media engagement, normative perceptions, and behavior

Virality metrics work as a key indicator of engagement (Kim, 2018) and message effectiveness (Alhabash et al., 2019) in social media, showing how popular a piece of information is in real-time (Metzger et al., 2010; Calabrese and Zhang, 2019). Theoretically, virality metrics are one type of online social informational cues that signify the number of users’ reactions to the information (e.g., the numbers of shares, Likes, and comments, Walther and Jang, 2012). Shares, Likes, and comments each tap into three components of virality (i.e., viral reach, affective evaluation, message deliberation): Shares tap ‘viral reach’ because users acknowledge the value of a given message by proactively forwarding it to their social networks; Likes involve ‘affective evaluation’ as users express their positive evaluation of a message, which becomes visible to other users; and comments tap ‘message deliberation’ because users deliberate on the information from a message in an active and public way (Alhabash and McAlister, 2015). In this study, virality metrics are crucial for demonstrating how users have engaged with the mask-wearing campaign message by health authorities.

Research has shown that virality metrics of a social media post can be a stronger predictor of further engagement than the information quality of the post (Weng et al., 2012). Serving as an endorsement heuristic (Sundar et al., 2009), virality metrics, for example, are found to correlate with higher numbers of sharing on social media (Aswani et al., 2017). Upon receiving a message with large virality metrics, people are inclined to perceive that the message is credible as others endorse and approve it (Metzger et al., 2010). People also tend to agree with the message under the bandwagon effect (Sundar et al., 2009), which may provoke their further engagement with it. Research on political polarization also showed that social endorsements as communicated through virality metrics can shape engagement behaviors including news selection (Messing and Westwood, 2014). Accordingly, we hypothesize that people are more likely to engage with the mask-wearing message with larger virality metrics on social media.

With many others’ approval of and support for the message, as signified with the large virality metrics, people may be more easily influenced by the information. This is supported by Lee-Won et al.’s (2017), which found a positive relation between virality metrics and the promotion of health information, specifically the intention to perform cancer screening *via* social media. In the context of alcohol consumption, the intention to consume alcohol was strongly predicted by the intention to engage with a social media message, especially when the message had high virality metrics (Alhabash et al., 2015). Virality metrics can be viewed as persuasive cues that systematically change people’s perceptions regarding certain behaviors (Calabrese and Zhang, 2019). This persuasive effect on behavioral intention can be theoretically identified as a normative influence that leads people to conform either consciously or unconsciously (Nolan et al., 2008; Calabrese and Zhang, 2019). While norms largely shape behaviors, injunctive norms, for instance, have been found to increase with virality metrics (Lee-Won et al., 2016), which then leads to a stronger intention to behave in line with the norms. Also, according to the Theory of Reasoned Action and Planned Behavior, subjective norms, the beliefs of whether most people approve the message or not, can predict behavioral intentions through social pressures (Ajzen, 1991). Thus, high virality metrics accompanying the mask-wearing message would imply heightened mask-wearing norms to ultimately encourage mask-wearing behavioral intention.

*H1*: High engagements, compared to low engagements, promote (a) social media engagement, and (b) mask-wearing norms and (c) behavioral intention.

### 3.2. The influence of opinion climate on social media engagement, normative perceptions, and behavior

Research has shown that a consensual opinion climate, compared to a mixed one, leads people to perceive a given piece of information more favorably, and further increase people’s likelihood of expressing an opinion (Duncan and Coppini, 2019). On the one hand, in a political context, for instance, individuals’ views are more quickly crystalized when their views are endorsed by their network; accordingly, they are more likely to express their views (Mutz, 2002). Thus, when participants who (are inclined to) support mask-wearing see comments consistent with their existing or emerging attitude, they may feel confident and show their support through further engagement with the mask-wearing campaign message, for instance, by posting pro-mask comments. On the other hand, according to the corrective action hypothesis, upon sensing that their view is opposed by their network, people may also express their views and participate in an effort to counteract the perceptions that are hostile to their viewpoint (Barnidge and Rojas, 2014). Relatedly, under the backfire effect, people may strengthen their misbeliefs after encountering corrective information although empirical results for this effect are mixed (Haglin, 2017; Nyhan and Reifler, 2019). In the context of the pandemic, presumed influence of social media COVID-19 misinformation on others provoked people’s willingness to support corrective actions (Luo and Cheng, 2021). In this study, when participants opposing mask-wearing saw comments supporting mask-wearing campaign messages, which they could perceive as misinformation, they were more likely to express their views with the urge to correct other users through further engagement. In both cases, we hypothesize that comments universally supporting the campaign messages will promote further social media engagement with the message.

Supporting comments can also be a salient endorsement heuristic. The endorsement indicated by supporting comments is a part of an aggregated valence of comments, operationalizing the opinion climate surrounding the message (Shi et al., 2022). It is reported that there is a consistency between the overall valence of opinions expressed in existing comments and people’s attitudes to the message (Hsueh et al., 2015; Sung and Lee, 2015). For instance, exposure to an opinion endorsing flu vaccines with favorable comments resulted in positive attitudes toward vaccines; also, a dominant number of supportive comments led to a greater perceived distribution of the supportive opinion (Kim et al., 2020). Furthermore, under the “Bandwagon effect,” people make choices based on their perceived consensus of others (Simon, 1954; Bass, 1969). In the social media context, while the perceived consensus can be captured with the dominant opinion climate, the choices people make can involve further engagement and behavioral intention. Alhabash et al. (2015) also found that people’s social media engagement is based on favorable evaluations of persuasive messages, and they are closer to the next step to perform offline behavior in line with the messages. Thus, supportive comments on the mask-wearing message may signify a supportive opinion climate to encourage further social media engagement from both sides, and lead people to make choices regarding mask-wearing norms and behavior that are consistent with the message.

*H2*: Comments supporting the wear-a-mask post, compared to mixed (including both supportive and opposing) comments, promote (a) social media engagement, and (b) mask-wearing norms and (c) behavioral intention.

### 3.3. Materials and methods

#### 3.3.1. Procedure

**Figure 3:**
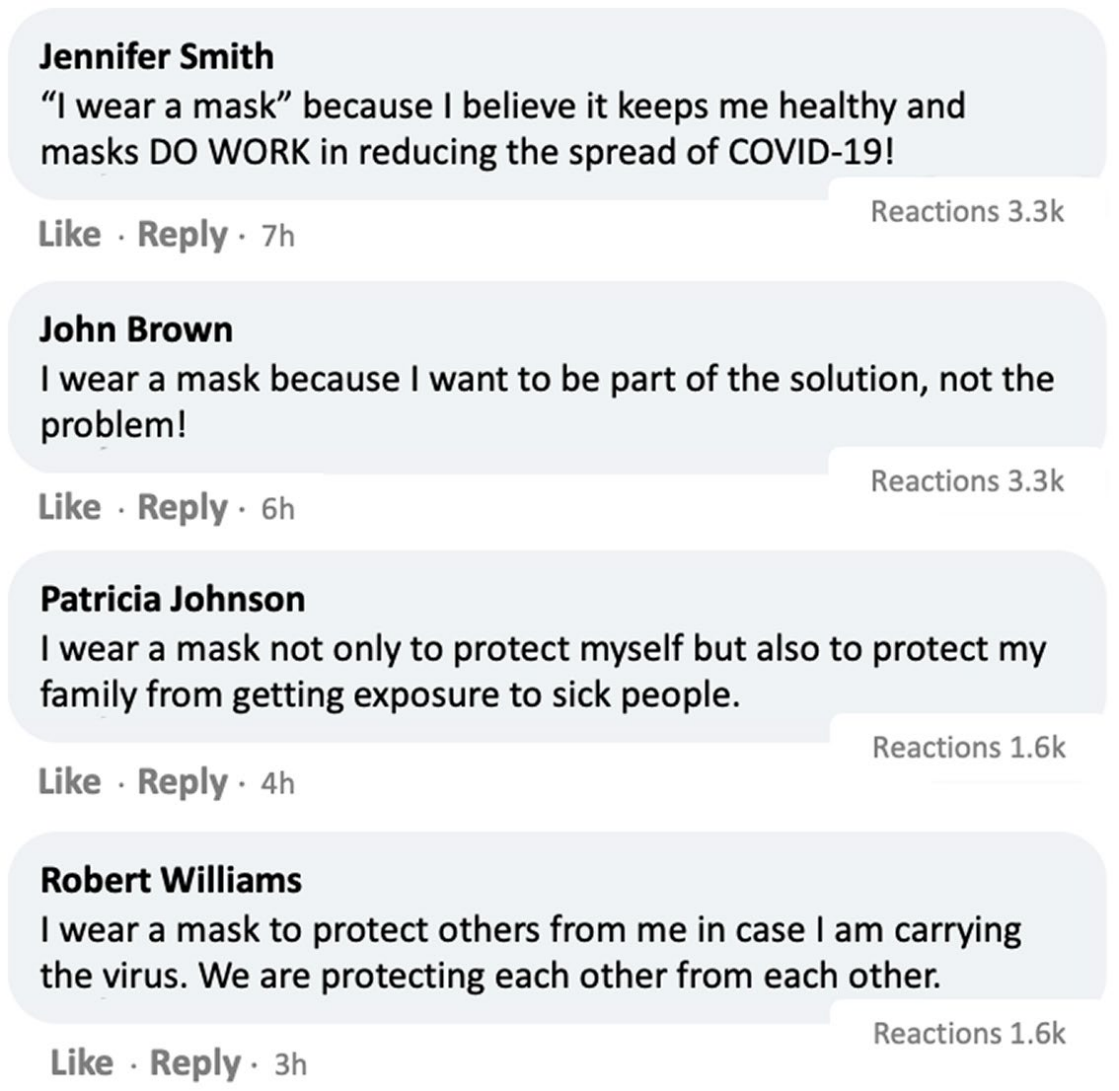
Experimental stimulus with high engagement and supportive comments. Note. In the place of “Reactions,” Facebook’s Like, Haha and love reaction emojis (https://about.meta.com/brand/resources/facebookapp/reactions/) were inserted.

**Figure 4:**
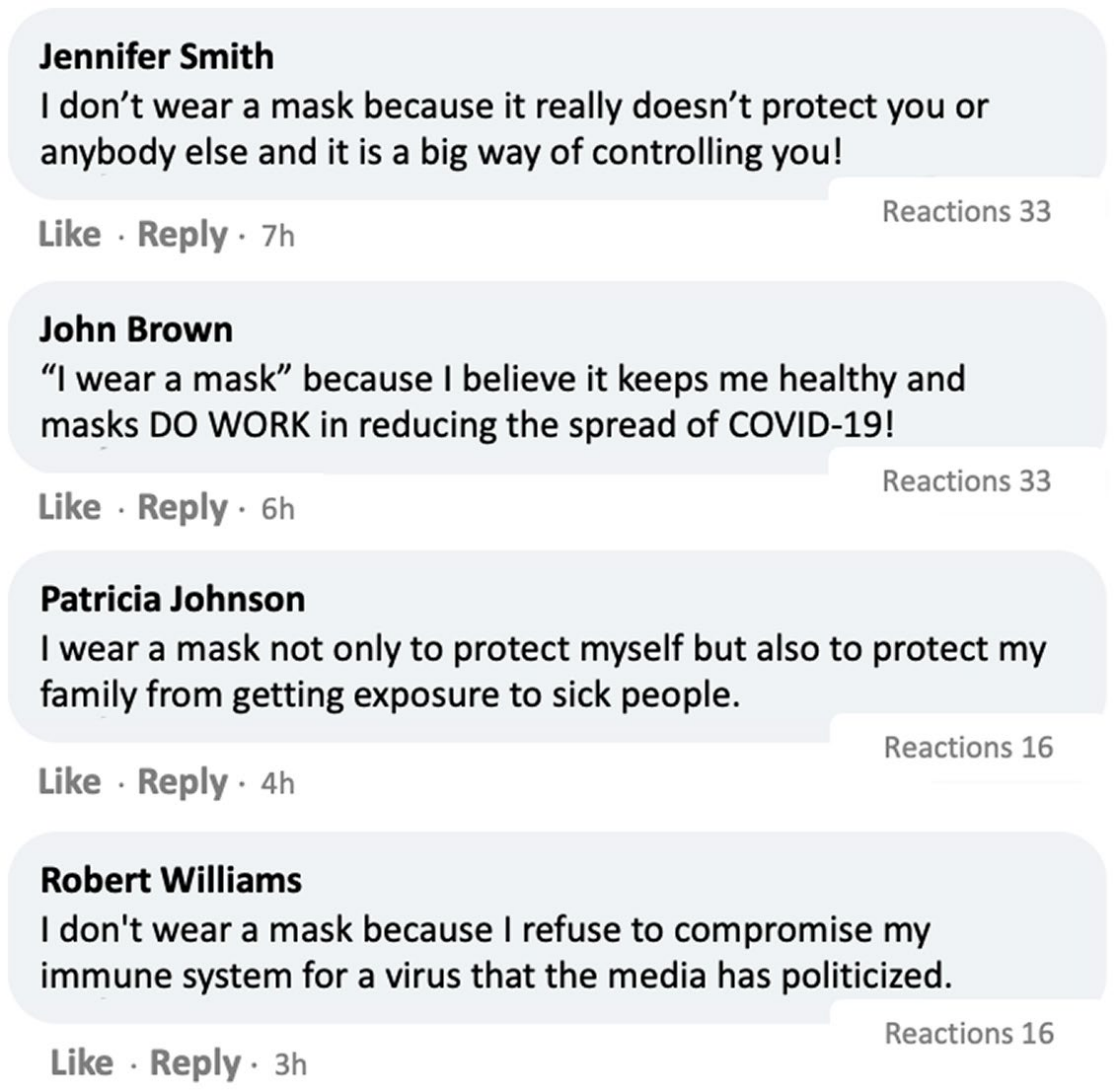
Experimental stimulus with low engagement and mixed comments. Note. In the place of “Reactions,” Facebook’s Like, Haha and love reaction emojis (https://about.meta.com/brand/resources/facebookapp/reactions/) were inserted.

Participants in the U.S. (*N* = 1,194) and in South Korea (*N* = 550) first read a prompt, “You will be shown a screenshot of a post by the CDC (or the DCPA in Korea) on a social media platform. Please read the post. You will be asked a few questions about it.” Then they were presented with one of the four stimuli with a CDC post encouraging mask-wearing,^3^ which varied by social media engagement metrics and the opinion climate of comments. Specifically, *social media engagement metrics* were manipulated by randomly assigning participants to two conditions where the provided CDC post showed a *high* (21.3 k reactions, 17.9 k comments, 12.8 k shares) or *low* (213 reactions, 179 comments, 128 shares) number of engagement metrics. The *opinion climate of comments* was manipulated through two conditions where they were exposed to four comments *supportive* of the CDC post (Figure 3) or *mixed* comments (i.e., two supportive and two opposing comments, Figure 4). Finally, respondents answered a series of post-treatment questions and were debriefed at the end.

#### 3.3.2. Measures

##### 3.3.2.1. Mask-wearing norms

Respondents indicated how true the following two statements were: a) Most Americans [Koreans] expect me to wear masks/face coverings and b) Most Americans wear masks/face coverings, on a 5-point scale, ranging from 1 (not at all true) to 5 (extremely true) (U.S.: *M* = 3.19, *SD* = 1.12, *r* = 0.76; Korea: *M* = 4.51, *SD* = 0.53, *r* = 0.65).

##### 3.3.2.2. Mask-wearing behavior

Respondents reported how true the following statement was: I intend to wear masks/face coverings within the next month, on a 5-point scale (U.S.: *M* = 3.35, *SD* = 1.51; Korea: *M* = 4.69, *SD* = 0.65).

##### 3.3.2.3. Social media engagement

Respondents indicated how likely the following three statements were: It is likely that I (a) share; (b) ‘Like’; and (c) comment on this post on social media. Response options ranged from 1 (not at all likely) to 5 (extremely likely). An index was created by averaging the three items (U.S.: *M* = 2.24, *SD* = 1.44, *a* = 0.96; Korea: *M* = 2.49, *SD* = 1.00, *a* = 0.86).

### 3.4. Results

#### 3.4.1. The U.S.

Hypothesis 1 predicted that high engagements, compared to low engagements, promote (a) social media engagement, and (b) mask-wearing norms and (c) behavior. To test hypotheses, three independent samples t-tests were conducted (Table 4). First, *social media engagement* numbers were not different between participants who were presented with a campaign message with high engagement numbers (*M* = 2.26, *SD* = 1.44) and those who were presented with the same message with low engagement numbers (*M* = 2.22, *S*D = 1.44), *t* = 0.49, *p* = 0.31 (H1a rejected). Second, *mask-wearing norms* were higher when participants were presented with a campaign message with high engagement numbers (*M* = 3.29, *S*D = 1.10) than that with low engagement numbers (*M* = 3.10, *S*D = 1.14), *t* = 3.04, *p* < 0.01 (H1b supported). Third, *mask-wearing intention* was higher to a marginal degree when participants were presented with a campaign message with high engagement numbers (*M* = 3.41, *S*D = 1.49) than that with low engagement numbers (*M* = 3.29, *S*D = 1.54), *t* = 1.39, *p* < 0.10. We find marginal support for H1c.

**Table 4:** Study 2: social media engagement and mask-wearing norms and intention as a function of high vs. low engagement (top) and supportive vs. mixed comments (bottom) in the U.S.

	High engagement		Low engagement			
	*M*	*SD*	*M*	*SD*	*t*	*p*
Social media engagement	2.26	1.44	2.22	1.44	0.49	0.31
Mask-wearing norms	3.29	1.10	3.10	1.14	3.04	<0.01
Mask-wearing intention	3.41	1.49	3.29	1.54	1.39	<0.10
	Supportive comments		Mixed comments			
	*M*	*SD*	*M*	*SD*	*t*	*p*
Social media engagement	2.35	1.49	2.14	1.38	2.45	<0.01
Mask-wearing norms	3.23	1.15	3.16	1.10	1.05	0.15
Mask-wearing intention	3.35	1.51	3.34	1.52	0.10	0.46

Next, Hypothesis 2 predicted that comments supporting the CDC post, compared to mixed comments, promote a) social media engagement, and b) mask-wearing norms and c) behavior. First, social media engagement numbers were higher when participants were presented with a campaign message with supportive comments (*M* = 2.35, *S*D = 1.49) than that with mixed comments (*M* = 2.14, *S*D = 1.38), *t* = 2.45, *p* < 0.01 (H2a supported). Second, mask-wearing norms were not different between participants who were presented with a campaign message with supportive comments (*M* = 3.23, *SD* = 1.15) and those who were presented with the same message with mixed comments (*M* = 3.16, *S*D = 1.10), *t* = 1.05, *p* = 0.15 (H2b rejected). Third, mask-wearing intention was not different when participants were presented with a campaign message with supportive comments (*M* = 3.35, *S*D = 1.51) than that with mixed comments (*M* = 3.34, *S*D = 1.52), *t* = 0.10, *p* = 0.46 (H2c rejected).

#### 3.4.2. South Korea

Regarding H1, first, social media engagement numbers were not different between participants who were presented with a campaign message with high engagement numbers (*M* = 2.48, *SD* = 1.04) and those who were presented the same message with low engagement numbers (*M* = 2.48, *S*D = 1.04) or that with low engagement numbers (*M* = 2.50, *S*D = 0.97), *t* = −0.31, *p* = 0.38 (Table 5, H1a rejected). Second, mask-wearing norms were higher when participants were presented with a campaign message with high engagement numbers (*M* = 4.56, *S*D = 0.54) than that with low engagement numbers (*M* = 4.47, *S*D = 0.51), *t* = 1.86, *p* < 0.05 (H1b supported). Second, mask-wearing intention was higher when participants were presented with a campaign message with high engagement numbers (*M* = 4.74, *S*D = 0.62) than that with low engagement numbers (*M* = 4.64, *S*D = 0.67), *t* = 1.80, *p* < 0.05 (H1c supported).

**Table 5:** Study 2: social media engagement and mask-wearing norms and intention as a function of high vs. low engagement (top) and supportive vs. mixed comments (bottom) in Korea.

	High engagement		Low engagement			
	*M*	*SD*	*M*	*SD*	*t*	*p*
Social media engagement	2.48	1.04	2.50	0.97	−0.31	0.38
Mask-wearing norms	4.56	0.54	4.47	0.61	1.86	<0.05
Mask-wearing intention	4.74	0.62	4.64	0.67	1.80	<0.05
	Supportive comments		Mixed comments			
	*M*	*SD*	*M*	*SD*	*t*	*p*
Social media engagement	2.54	1.06	2.44	0.96	1.22	0.11
Mask-wearing norms	4.53	0.53	4.50	0.52	0.82	0.21
Mask-wearing intention	4.66	0.74	4.71	0.55	−1.00	0.16

Next, with regard to H2, first, social media engagement not different between participants who were presented with a campaign message with supportive comments (*M* = 2.54, *SD* = 1.06) and those who were presented with the same message with mixed comments (*M* = 2.44, *S*D = 0.96), *t* = 1.22, *p* = 0.11 (H2a rejected). Second, mask-wearing norms were not different between participants who were presented with a campaign message with supportive comments (*M* = 4.53, *SD* = 0.53) and those who were presented with the same message with mixed comments when participants were presented with a campaign message with supportive comments (*M* = 4.53, *S*D = 0.53) than that with mixed comments (*M* = 4.50, *S*D = 0.52), *t* = 0.82, *p* = 0.21(H2b rejected). Finally, mask-wearing intention not different participants who were presented with a campaign message with supportive comments (*M* = 4.66, *SD* = 0.74) and those who were presented with the same message with mixed comments.

## 4. Discussion

Social media can be a double-edged sword for public health by making health information accessible to a wider population while also aiding in the spread of incorrect or misleading information or views against crucial public health behaviors (Huber et al., 2019; Kouzy et al., 2020; Chou et al., 2021). During global health crises, such as the COVID-19 pandemic with extreme uncertainty and health risks, it is essential that we have a good theoretical understanding of how preventive health behaviors and norms can be fostered on social media to make the best use. For this, the current study analyzed data from surveys (Study 1) and experiments (Study 2) conducted in two different contexts: the U.S. and South Korea. Study 1 findings suggest a pathway from social media use for COVID-19 information to mask-wearing behavior through mask-wearing norms, consistent with the Theory of Reasoned Action and Planned Behavior (Ajzen, 1991). Importantly, this pathway emerges only among individuals with strong perceived social media literacy in the U.S. Study 2 presents experimental evidence that wear-a-mask campaign posts by health authorities on social media can strengthen mask-wearing norms and behavioral intention when they are accompanied with large virality metrics such as Likes, comments and shares, across the U.S. and Korea. Overall, the results underscore the potential of social media as a space for the nurturing of preventive health behaviors that depend on collective coordination such as masking, through shaping of norms. Study 1 results additionally suggest this is especially true for users who have greater sense of social media literacy.

### 4.1. Study 1: the roles of perceived norms and social media literacy

Study 1 finds support for the moderated mediation model in which social media use for COVID-19 information can foster mask-wearing norms and behaviors among users with strong perceived social media literacy in the U.S. (Figure 1). Considering that various groups, such as racial minorities, younger people and possibly less educated individuals, tend to engage in more health-related activities on social media (Chou et al., 2021), this result highlights the potential of social media to be beneficially used for health information and to help with closing the digital divides. However, our findings also note that individuals may not enjoy these benefits if they do not perceive themselves competent in using social media. Indeed, health and COVID-19-related misinformation is rife on social media (Kouzy et al., 2020; Stecula et al., 2020). With false information presented along with correct information on social media feeds, users need to be confident with their literacy skills in order to critically process misinformation and be properly informed (Borah and Xiao, 2018; Vraga et al., 2021).

Indeed, we should recognize that social media content is diverse. Does exposure to all types of COVID-19 content result in the same findings? We were able to conduct further analysis to probe this follow-up question. Our index of social media use for COVID-19 information consisted of three items, two items on content from lay individuals and one item from news sources and public figures. Post-hoc tests revealed that the moderated mediation relationship was held regarding content from lay people (*b* = 0.03, SE = 0.01, 95% CI [0.007, 0.061]), meaning that mask-wearing norms and behaviors were strengthened after consumption of content and opinions from lay people, only among those with strong perceived social media literacy. However, the index of moderated mediation did not reach statistical significance (*b* = 0.03, SE = 0.01, 95% CI [−0.002, 0.053]) regarding content from news sources and public figures although the interaction and mediation (*b* = 0.04, SE = 0.02, 95% CI [0.004, 0.078]) were significant. That is, all users, regardless of their perceived social media literacy, reported heightened mask-wearing norms and behaviors after exposure to COVID-19 content from news sources and public figures. Perhaps social media literacy is especially useful for critical processing of information from lay people, rather than official sources or experts. On the practical front, to benefit all social media users, it would be important to widen the reach of content by health authorities and experts. Aiding people distinguish between content from official sources and lay people is also crucial, as it relates to their social media literacy.

### 4.2. Study 2: the importance of virality metrics accompanying campaign messages

Social media have the potential to promote health authorities’ campaigns because they can provide widened reach with lower costs, tailored messages and heightened interactivity (Chou et al., 2013). Focusing on the interactivity afforded on social media, Study 2 presents experimental evidence that wear-a-mask campaign posts by the CDC can effectively promote mask-wearing norms and behavioral intention with the aid of large virality metrics (e.g., Likes, comments and shares) rather than supportive comments in both countries. This is in line with prior studies viewing virality metrics as an endorsement heuristic; under the bandwagon effect, individuals tend to perceive that a message with large virality metrics is approved by many others, and thus think it is credible and adoptable (Sundar et al., 2009; Borah and Xiao, 2018). A message coming with large virality metrics can be considered credible, often regardless of whether the underlying comments are supportive or mixed (Metzger et al., 2010).

In Study 2, as endorsement heuristics, the large virality metrics accompanying the CDC’s posts can signify that many others endorse mask-wearing, thereby strengthening mask-wearing norms, which may then lead to a stronger intention to behave consistent with the norms. Thus, for successful campaigns, health authorities and experts may encourage social media users to actively engage with their messages by sharing, reacting to and commenting on them. While our results show that large virality metrics can foster norms and behaviors in line with the campaign messages among users, social media algorithms will likely further rank these messages higher to reach even more users through positive feedback loops. Although supportive comments, compared to mixed ones, were not found to strengthen mask-wearing norms and behavioral intention, they may facilitate further social media engagement (e.g., Like, share and comment). In a way, supportive comments can effectively help with building larger vitality metrics for successful campaign messages.

On a practical level, these findings highlight the potential value of boosting pro-mask wearing content from official sources in ways that target increased organic engagement from users. At a broader level, it shows the public health value of content regulation in social media that promotes health messages. At the same time, messaging interventions targeting user metrics on social media should be carefully implemented with strong transparency and the coordination of multiple stakeholders, including members of the public, to prevent pitfalls of (or backlash against perceived) social engineering (Freiling et al., 2023).

### 4.3. Cross-national differences between the U.S. and Korea

The current study examined all the hypotheses and research questions in two very different empirical contexts, the U.S. and South Korea, and investigated how their contextual difference might matter for our empirical results (see Esser, 2014). There are a few reasons why this comparative investigation is important: First, the pandemic severity and management by governments have been different across countries. The U.S. suffered significantly more cases and fatalities during the pandemic, and mask wearing rules in public places have been more contentious with significant variations across different states and localities (Bromwich, 2020). Second, two societies differ in terms of individualistic vs. collectivistic cultures. According to the Culture Compass survey, the U.S. scores much higher than Korea on individualism while Korea scores much higher than the U.S. on long term orientation dimension (Hofstede Insights, 2022). This cultural difference may be an important factor for how group norms and collective action can influence behaviors such as mask wearing in the context of public health management and media use (see Kim and Kwak, 2021).

In Study 1, we found support for the pathway from social media use for COVID-19 information to mask-wearing behavior through norms, which was moderated by perceived social media literacy, only in the U.S.^4^ Perhaps this was due to ceiling effects; the mean score of mask-wearing behavior in Korea was 4.69, close to the highest point, 5, while that in the U.S. was 3.35. Also, the standard deviation of mask-wearing norms in Korea was low, 0.53, possibly reflecting Korea’s strong collective norms of mask-wearing. This may be due to the two countries’ different mask mandates: During the data collection period, in the U.S., fully vaccinated people were no longer required to wear masks under the federal guidance, with varying local guidelines (Netburn, 2021). However, Koreans, even fully vaccinated ones, were required to wear masks indoors as well as outdoors most of the times (DCPA, 2021). Even before the pandemic, the use of masks was considered polite when people were sick in Asia including Korea; Asia also experienced the 2002–3 SARS-1 outbreak which was caused by another coronavirus, and this historical memory may make mask wearing more easily acceptable (Wong, 2020). In Study 2, the results were largely consistent across the two countries, speaking to the applicability of the experimental findings in both societies.^5^

### 4.4. Limitations and future research directions

Despite its contributions, the current study bears a few limitations. First, Study 1 analyzed cross-sectional survey data, and caution needs to be taken in drawing causal inferences. Although the moderated mediation model was theoretically driven, we tested for the possibility of reverse causality. No support was found for reverse causality,^6^ granting more confidence in the results. Relatedly, since Study 1 relied on self-reports, measures such as social media use may not have been the most ideal ones. Also, perceived social media literacy might have been inflated with an overconfidence bias. Future research should measure social media literacy using a variety of questions to explore other dimensions. Still, self-reports effectively captured other main variables including norms.

Next, Study 2 analyzed experimental data, and thus its internal validity is high while its external validity is not. For instance, participants were exposed to screenshots of the health authorities’ campaign posts with four comments by strangers, which could feel artificial. This holds true although it was a strategic methodological decision by the authors to combine evidence from surveys and experiments for a more comprehensive understanding of how mask-wearing norms and behaviors can be fostered on social media. Also, the current study investigated mask-wearing as one preventive health behavior, potentially limiting the generalizability of its results.

Finally, future research extending the current study may collect data from more longitudinal surveys along with carefully designed experiments with less artificial settings of social media to more confidently draw causal conclusions. Also, it would be worthwhile to examine a wider variety of preventive health behaviors beyond mask-wearing for generalizability. Lastly, self-reported measures of social media use and literacy can be complemented with social media log data and literacy tests.

## Data availability statement

The raw data supporting the conclusions of this article will be made available by the authors, without undue reservation.

## Ethics statement

The study conducted in the U.S. was determined exempt after a review by the Institutional Review Board of the University of Arizona. The study conducted in Korea was reviewed and approved by the Institutional Review Board of Korea University. The participants provided their written informed consent to participate in this study.

## Author contributions

DK, OK, and SK contributed to conception and design of the study, and data analysis. DK and SK collected data, acquired funding, and edited the manuscript. DK wrote the first draft of the manuscript. OK and JZ wrote sections of the manuscript. All authors read and approved the submitted version.

## Funding

This work was supported by the Ministry of Education of the Republic of Korea, the National Research Foundation of Korea (NRF-2019S1A3A2099973), the MSIT (Ministry of Science and ICT) Korea, under the ITRC (Information Technology Research Center) support program (IITP-2020-0-01749) supervised by the IITP (Institute of Information & Communications Technology Planning & Evaluation), and the University of Arizona.

## Conflict of interest

The authors declare that the research was conducted in the absence of any commercial or financial relationships that could be construed as a potential conflict of interest.

## Publisher’s note

All claims expressed in this article are solely those of the authors and do not necessarily represent those of their affiliated organizations, or those of the publisher, the editors and the reviewers. Any product that may be evaluated in this article, or claim that may be made by its manufacturer, is not guaranteed or endorsed by the publisher.

## Footnotes

^1^Both surveys were conducted during the COVID third wave when the Delta variant surged. The daily average cases in the U.S. were about 12,000 (0.004% of population); Korea had about 1,400 new cases daily (0.003% of population). The data collection dates and sample sizes differed between the two countries due to logistical issues and funding complications. However, this is not expected to influence results because there were no substantive events in Korea during these 2 months. We also replicated the findings using a randomly selected subsample of the American sample to match the Korean sample size (see Footnote 3).

^2^Item-by-item *post-hoc* analysis is reported in Section 4.

^3^The CDC post in the stimuli contained the same text from this post by CDC on Facebook about the wear-a-mask campaign (https://www.facebook.com/watch/?v=858138214980932) while in the place of a videoclip, a screenshot of this video by CDC at 0:06 (https://www.facebook.com/watch/?v=255992979505443) was inserted. In all four conditions, the same textual message and image were shown.

^4^Also, the link between social media for COVID-19 information and mask-wearing norms was positive only in the U.S. The measure of mask-wearing norms is a combination of injunctive and descriptive norms, which tap how socially approved and popular an act is, respectively. Americans who heavily used social media to get information about COVID-19 may have been able to think of mask-wearing instances easily (i.e., availability heuristics, Folkes, 1988) to consider it socially approved and widely performed.

^5^There were two minor differences: In the U.S., mask-wearing intention increased with large (vs. small) virality metrics to a marginal degree while in Korea, the difference was statistically significant. In the U.S., social media engagement increased with supportive (vs. mixed) comments while in Korea, the difference was not significant. When we tested the hypotheses in the U.S. using a randomly selected subsample whose size was equivalent to the Korean sample, the results were mostly the same. However, mask-wearing intention no longer increased with large (vs. small) virality metrics (*t* = 0.23 *p* > 0.10) and social media engagement increased with supportive (vs. mixed) comments to a marginal degree (*t* = 1.45, *p* < 0.10).

^6^When we test for the alternative moderated mediation model where social media use for COVID-19 information is switched with mask-wearing behavior, and perceived social media literacy moderates the link between mask-wearing norms and social media use, the model is no longer significant in the U.S. (*b* = 0.01, SE = 0.01, 95% CI [−0.015, 0.042]).
